# Understanding the relationship between circulating lipids and risk of chronic kidney disease: a prospective cohort study and large-scale genetic analyses

**DOI:** 10.1186/s12967-023-04509-5

**Published:** 2023-09-27

**Authors:** Yutong Wang, Li Zhang, Wenqiang Zhang, Mingshuang Tang, Huijie Cui, Xueyao Wu, Xunying Zhao, Lin Chen, Peijing Yan, Chao Yang, Chenghan Xiao, Yanqiu Zou, Yunjie Liu, Ling Zhang, Chunxia Yang, Yuqin Yao, Jiayuan Li, Zhenmi Liu, Xia Jiang, Ben Zhang

**Affiliations:** 1 Department of Epidemiology and Biostatistics, Institute of Systems Epidemiology, and West China-PUMC C. C. Chen Institute of Health, West China School of Public Health and West China Fourth Hospital, Sichuan University, No. 16, Section 3, South Renmin Road, Wuhou District, Chengdu, 610041 Sichuan China; 2 Department of Maternal, Child and Adolescent Health, West China School of Public Health and West China Fourth Hospital, Sichuan University, Chengdu, China; 3 Department of Iatrical Polymer Material and Artificial Apparatus, School of Polymer Science and Engineering, Sichuan University, Chengdu, China; 4 Department of Occupational and Environmental Health, West China School of Public Health and West China Fourth Hospital, Sichuan University, Chengdu, China; 5 Department of Nutrition and Food Hygiene, West China School of Public Health and West China Fourth Hospital, Sichuan University, Chengdu, China; 6 Department of Clinical Neuroscience, Karolinska Institutet, Stockholm, Sweden

**Keywords:** Lipids, Chronic kidney disease, Kidney function, Genetic correlation, Genome-wide cross-trait analysis, Mendelian randomization

## Abstract

**Background:**

This study aims to comprehensively investigate the phenotypic and genetic relationships between four common lipids (high-density lipoprotein cholesterol, HDL-C; low-density lipoprotein cholesterol, LDL-C; total cholesterol, TC; and triglycerides, TG), chronic kidney disease (CKD), and estimated glomerular filtration rate (eGFR).

**Methods:**

We first investigated the observational association of lipids (exposures) with CKD (primary outcome) and eGFR (secondary outcome) using data from UK Biobank. We then explored the genetic relationship using summary statistics from the largest genome-wide association study of four lipids (N = 1,320,016), CKD (N_case_ = 41,395, N_control_ = 439,303), and eGFR(N = 567,460).

**Results:**

There were significant phenotypic associations (HDL-C: hazard ratio (HR) = 0.76, 95%CI = 0.60–0.95; TG: HR = 1.08, 95%CI = 1.02–1.13) and global genetic correlations (HDL-C: ${r}_{g}$ = − 0.132, *P* = 1.00 × 10^–4^; TG: ${r}_{g}$ = 0.176; *P* = 2.66 × 10^–5^) between HDL-C, TG, and CKD risk. Partitioning the whole genome into 2353 LD-independent regions, twelve significant regions were observed for four lipids and CKD. The shared genetic basis was largely explained by 29 pleiotropic loci and 36 shared gene-tissue pairs. Mendelian randomization revealed an independent causal relationship of genetically predicted HDL-C (odds ratio = 0.91, 95%CI = 0.85–0.98), but not for LDL-C, TC, or TG, with the risk of CKD. Regarding eGFR, a similar pattern of correlation and pleiotropy was observed.

**Conclusions:**

Our work demonstrates a putative causal role of HDL-C in CKD and a significant biological pleiotropy underlying lipids and CKD in populations of European ancestry. Management of low HDL-C levels could potentially benefit in reducing the long-term risk of CKD.

**Graphical Abstract:**

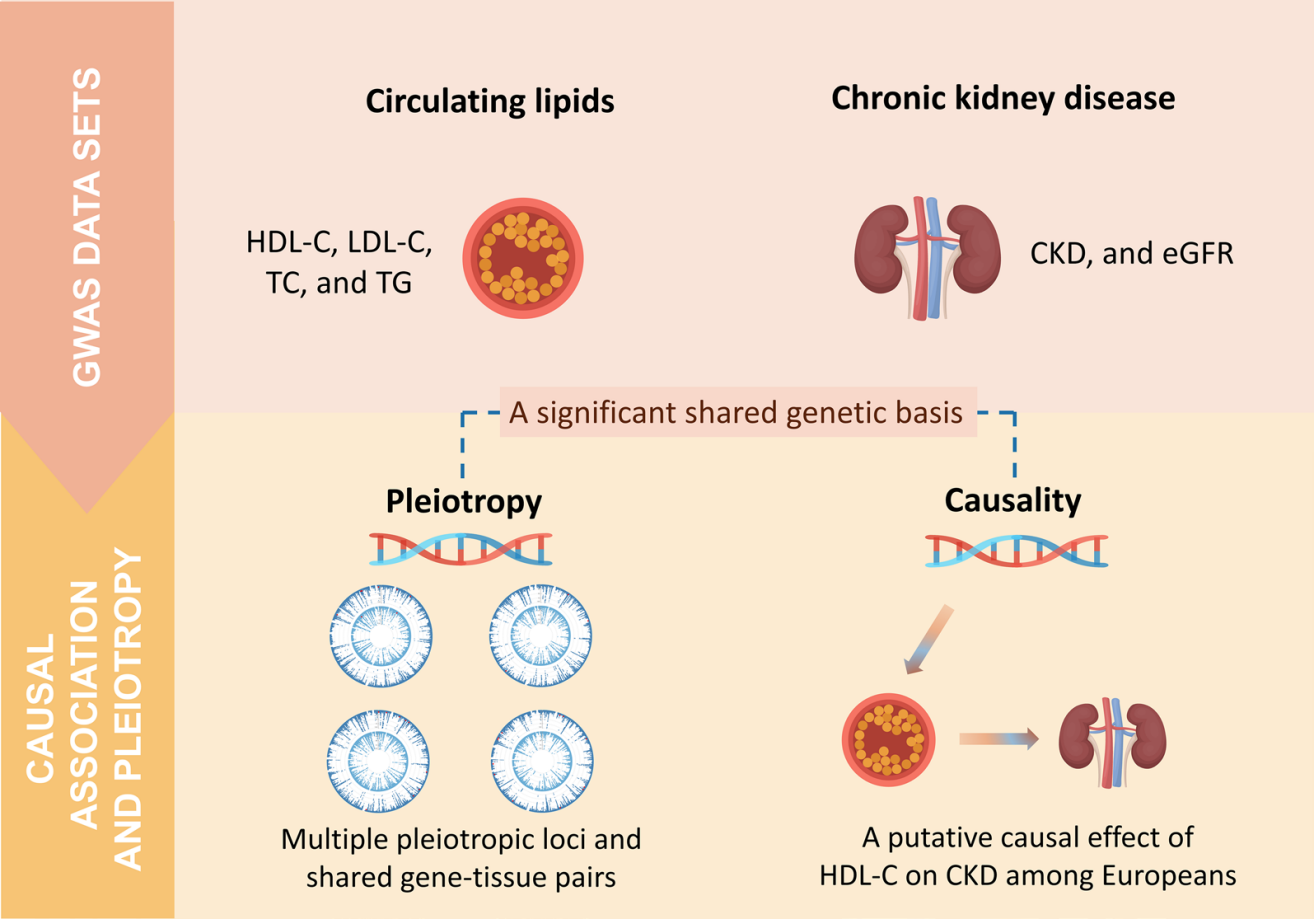

**Supplementary Information:**

The online version contains supplementary material available at 10.1186/s12967-023-04509-5.

## Introduction

Circulating lipids play an important yet complex role in the development of chronic kidney disease (CKD). While large-scale prospective cohort studies have demonstrated the risk effect of triglycerides (TG) (risk ratio (RR) = 1.28, 95%CI = 1.16–1.41) [1], low high-density lipoprotein cholesterol (HDL-C) (RR = 1.21, 95%CI = 1.12–1.30) [1], and low-density lipoprotein cholesterol (LDL-C) (RR = 1.04, 95%CI = 1.02–1.06) [2] on the onset of CKD, these findings have not been supported by clinical studies aimed at understanding the effects of lipid-lowering therapies. Recent clinical trials have shown little benefit or even deleterious effect among treated dyslipidemia patients in relation to incident kidney disease [3, 4]. Indeed, interventions in randomized trials are often implemented over relatively short periods, typically around 40 months for phase III trials [5]. Additionally, conventional observational studies can be susceptible to environmental confounding and reverse causality [6].

In the subsequent efforts raised to address these discrepancies, genetic data has been used to overcome these drawbacks. Notably, a modest genetic correlation underlying circulating lipids and CKD has been quantified by a twin study (TG-CKD, ${r}_{g}$ = 0.13; HDL-C-CKD, ${r}_{g}$ = -0.14) [7]. Multiple pleiotropic loci (i.e., *CD36* and *PCSK9*) have further been identified as affecting LDL-C and total cholesterol (TC) levels as well as CKD or kidney function [8–10]. Moreover, Mendelian randomization (MR) studies have suggested a putative causal effect of HDL-C on CKD, with estimates ranging from 0.85 to 0.96 [11–13]. Despite these progresses, there are still a few major gaps that remain to be addressed. First, except for HDL-C, the causal roles of other lipids on CKD remain unclear. While some MR studies reported significant causal effects of three lipids (LDL-C, TC, and TG) on CKD or kidney function [2, 14, 15], the others did not [12, 13]. Second, previous genetic research has relied on genome-wide association studies (GWAS) conducted on relatively small samples [2, 11–15], which limits the statistical power. Third, the majority of MR studies have not taken into account the independent effects of each lipid phenotype [12–14]. Fourth, the information obtained from previous genetic studies was relatively fragmented, lacking large-scale observational or genetic analyses that simultaneously investigate the degree and nature of shared etiology.

**Fig. 1:**
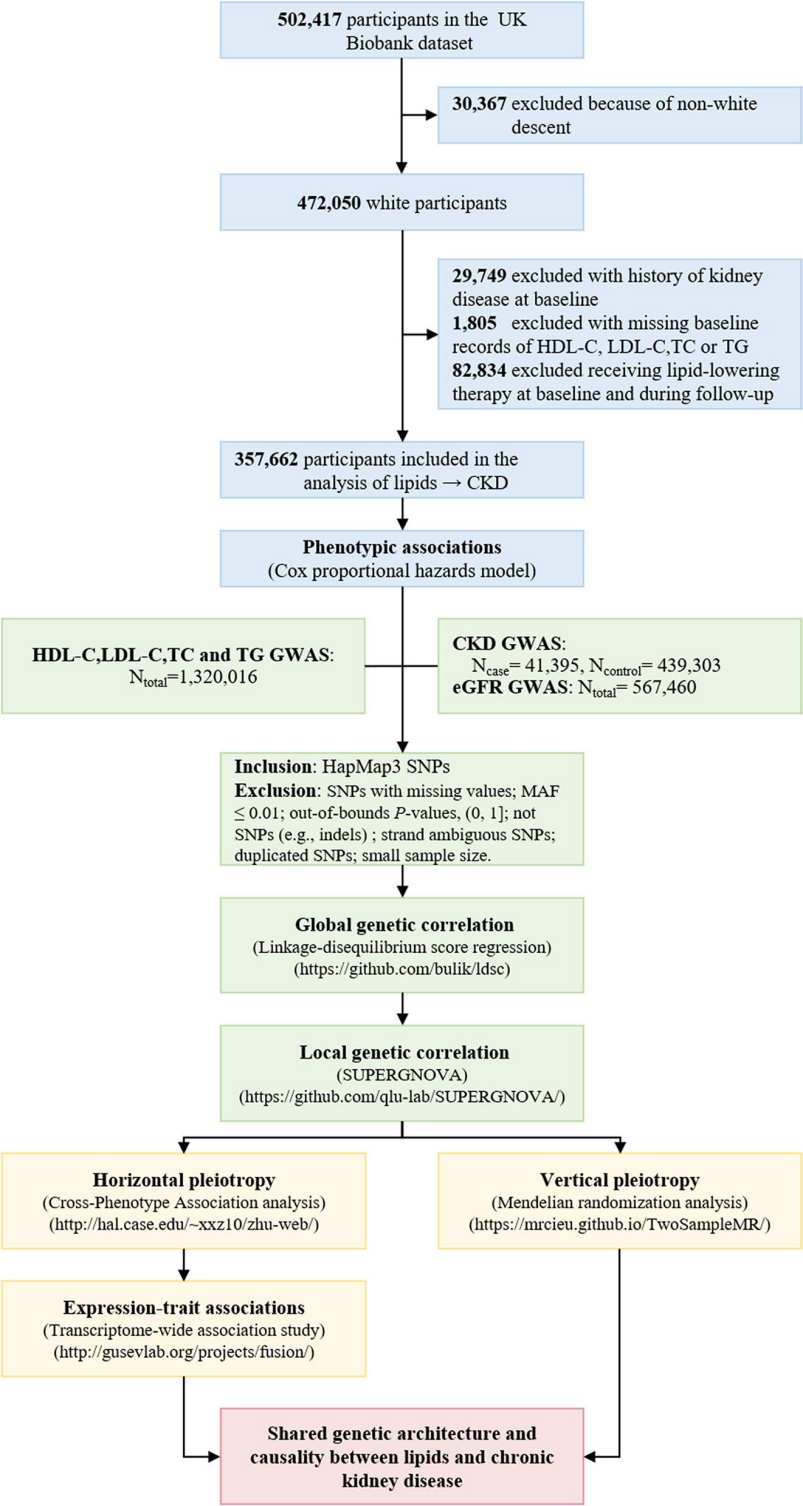
Flowchart of overall study design. HDL-C, high-density lipoprotein cholesterol; LDL-C, low-density lipoprotein cholesterol; TC, total cholesterol; TG, triglycerides; CKD, chronic kidney disease; eGFR, estimated glomerular filtration rate

Therefore, leveraging the hitherto largest observational and genetic data, we aimed to comprehensively evaluate the relationships between four lipid phenotypes (HDL-C, LDL-C, TC, and TG) with CKD and with estimated glomerular filtration rate (eGFR). We first examined the phenotypic association using individual-level data from the UK Biobank (UKB). We next performed genetic analyses to quantify global and local genetic correlations, identify pleiotropic loci, and detect putative causal relationships underlying these complex traits. We set CKD as the primary outcome of interest and eGFR as the secondary outcome. The overall study design is shown in Fig. 1.

## Methods

UK Biobank data UK Biobank is a large population-based prospective cohort study with over 500,000 individuals aged 40–69 at the time of recruitment in the UK from 2006 to 2010 [16]. All participants provided written informed consent, and UK Biobank received ethical approval from the National Health Service Research Ethics Service. We only considered participants of white British descent (N = 472,050). We defined CKD by the *International Classification of Diseases, tenth Revision (ICD-10)* code N18. We excluded participants with a history of kidney disease at baseline (ICD-9 codes 580–589; ICD-10 codes N00-N29), those with missing information of HDL-C, LDL-C, TC, or TG at baseline, or those receiving lipid-lowering therapy at enrollment and during follow-up, leaving 357,662 participants for the current analysis.

### GWAS summary statistics for lipids and CKD

Lipids GWAS The hitherto largest lipids GWAS summary statistics were obtained from the Global Lipids Genetics Consortium, aggregating 146 cohorts totaling 1,320,016 individuals of European ancestry [10]. We selected 380, 403, 429, and 388 single nucleotide polymorphisms (SNPs) of genome-wide significance (*P* < 5 × 10^–8^) as instrumental variables (IVs) for HDL-C, LDL-C, TC, and TG, respectively (Additional file 1: Tables S1–S4). We also applied GWAS summary statistics for other analyses.

CKD GWAS The hitherto largest GWAS of CKD was conducted by the CKDGen Consortium, meta-analyzing data from 23 participating studies combining 41,395 cases and 439,303 healthy controls (all of European ancestry) [9]. CKD was defined as an eGFR below 60 ml/min per 1.73 m^2^. As the original GWAS did not report index variants, we thus selected 27 independent genome-wide significant (*P* < 5 × 10^–8^) SNPs by applying clumping at a linkage disequilibrium (LD) threshold of *r*^2^ < 0.01 (Additional file 1: Table S5). The relevant GWAS of eGFR comprised 567,460 individuals of European ancestry [9]. We selected 256 SNPs as IVs to proxy eGFR (Additional file 1: Table S6). To minimize the false positive findings due to sample overlap, we further adopted another CKD GWAS for MR sensitivity analysis from the FinnGen consortium (https://r7.finngen.fi/), involving 6,604 cases and 299,094 controls.

### Statistical analyses

#### Observational analysis

Baseline characteristics of participants from the UK biobank were described as mean ± standard deviation (SD) for continuous variables and count (percentage) for discrete variables. We constructed a Cox proportional hazards regression model with baseline-measured HDL-C, LDL-C, TC, and TG as the exposure. We used three sets of adjustments. Estimates in model 1 (basic model) were adjusted for age, sex, region, body mass index (BMI), hypertension, diabetes mellitus, and the top 10 genetic principal components. Estimates in model 2 were further adjusted for income, Townsend deprivation index, smoking, drinking, physical activity, sleep duration, antihypertensive medications, and hypoglycemic medications usage at baseline and during follow-up. Estimates in model 3 (full model) were further adjusted for lipid fractions. In the sensitivity analysis, we excluded participants with less than a year of follow-up or a diagnosis of CKD within one year after enrollment. We further divided plasma lipids into quartiles and repeated the above analysis. All statistical analyses were performed using SAS version 9.4 (SAS Institute, Cary, NC). Statistical tests were two-sided, and significant levels were set at *P* < 0.05.

#### Genome-wide genetic correlation analysis

We quantified the genome-wide genetic correlation using LD score regression (LDSC) [17]. The genetic correlation estimates ${r}_{g}$ range from − 1 to + 1, with + 1 denoting a complete positive correlation and − 1 indicating a perfect negative correlation. Bonferroni correction was applied to correct for multiple statistical tests. We defined a significant ${r}_{g}$ as *P* < 6.25 × 10^−3^ (α = 0.05/8, number of phenotype pairs) and suggestive ${r}_{g}$ as 6.25 × 10^−3^ ≤ *P* < 0.05.

We further estimated the pairwise local genetic correlation using SUPERGNOVA [18]. This algorithm partitions the whole genome into 2,353 approximate LD-independent blocks and quantifies shared local effects driven by genetic variants at each particular region. A Bonferroni-adjusted P-threshold (*P* < 2.12 × 10^–5^ = 0.05/2,353) was applied to determine statistical significance.

#### Cross-trait meta-analysis

We next conducted a Cross-Phenotype Association (CPASSOC) analysis to identify potential pleiotropic variants that affect both traits [19]. We computed pairwise S_Het_, a statistic that is more powerful when heterogeneity exists. We used the PLINK clumping function to obtain independent shared variants (parameters: -clump-p1 5e-8 -clump-p2 1e-5 -clump-r2 0.1 -clump-kb 500) [20]. Significant index SNP was defined as *P*_CPASSOC_ < 5 × 10^–8^ and *P*_single-trait_ < 1 × 10^–5^ (for both traits). Novel shared SNP was defined if all following conditions were satisfied: (1) the SNP did not reach genome-wide significance (5 × 10^–8^ < *P*_single-trait_ < 1 × 10^–5^) in original single-trait GWAS; (2) the SNP was not in LD (*r*^2^ < 0.1) with any of those previously reported genome-wide significant SNPs in single-trait GWAS. Ensemble Variant Effect Predictor [21] was applied for detailed functional annotation of the identified pleiotropic SNPs.

#### Fine mapping credible-set analysis and colocalization analysis

We performed a fine-mapping analysis using FM-summary [22] to identify a credible set of SNPs 99% likely to contain the causal SNP at each of the shared loci obtained from CPASSOC. We conducted a colocalization analysis applying Coloc to calculate the posterior probabilities under the hypothesis of the sharing causal variants in a genomic region (H0-H4) [23]. A locus was considered colocalized if the posterior probability for H4 (PPH4, the probability that both traits are associated through sharing a single causal variant) was greater than 0.5.

#### Transcriptome-wide association studies

We performed a transcriptome-wide association study (TWAS) using FUSION based on 49 GTEx (version 8) tissue expression weights to investigate gene expression in specific tissues [24]. We first performed 49 TWASs for each trait, one tissue-trait pair at a time. We then conducted joint/conditional tests for a locus with multiple associated features to investigate conditionally independent genes at each locus. We further integrated single-trait TWAS results to identify shared gene-tissue pairs common to both traits.

#### Mendelian randomization analysis

Two-sample MR analyses were performed using summary statistic data to investigate the causal relationship. The inverse-variance weighted (IVW) approach was applied as the primary method [25]. As a complement to IVW, we also conducted MR-Egger regression and a weighted median approach [26, 27]. A Bonferroni-adjusted P-threshold of 6.25 × 10^−3^ (α = 0.05/8, number of phenotype pairs) was applied, and 6.25 × 10^−3^ ≤ *P* < 0.05 was defined as suggestive significance. An effect estimate was considered putative causal if it was significant in IVW and showed directional consistency in the MR-Egger regression and weighted median approach. We conducted an additional sensitivity analysis by excluding palindromic IVs with strand ambiguity and replicating the significant associations using nonoverlapped data. We further performed a multivariable MR (MVMR) approach to disentangle the independent effect of each lipid exposure while controlling for genetic predisposition to the remaining lipids traits, type 2 diabetes (T2D)[28], hypertension [16], and BMI [29]. Finally, we calculated *F-statistic* [30] for each set of IVs to assess weak instrument bias (Additional file 1: Table S7).

## Results

### Phenotypic association

The baseline characteristics of UKB participants included in the observational analysis were presented in Additional file 1: Table S8. In total, participants were followed for 4,342,332 person-years (12.14 ± 1.87 years), during which 7,934 individuals developed CKD (Table 1). In basic model, all four lipids were significantly associated with the risk of CKD (HDL-C: hazard ratio (HR) = 0.50, 95%CI = 0.46–0.54; LDL-C: HR = 0.90, 95%CI = 0.87–0.93; TC: HR = 0.89, 95%CI = 0.87–0.92; TG, HR = 1.13, 95%CI = 1.11–1.16). With further adjustments, significant associations remained only for HDL-C (HR = 0.68, 95%CI = 0.61–0.75) and TG (HR = 1.10, 95%CI = 1.06–1.13), while LDL-C or TC was no longer associated with CKD. In full model, the effect of HDL-C (HR = 0.76, 95%CI = 0.60–0.95) and TG (HR = 1.08, 95%CI = 1.02–1.13) attenuated to some extent yet remained statistically significant. Similar results were observed in the sensitivity analysis. The patterns remained consistent when each lipid variable was categorized into quartiles (Additional file 1: Table S9).

**Table 1:** Observational associations between lipids and subsequent risk of chronic kidney disease

	Primary analysis			Sensitivity analysis
	Model 1	Model 2	Model 3	
HDL-C	0.50(0.46–0.54)	0.68(0.61–0.75)	0.76(0.60–0.95)	0.78(0.62–0.99)
LDL-C	0.90(0.87–0.93)	0.97(0.94–1.01)	0.99(0.77–1.26)	1.01(0.79–1.29)
TC	0.89(0.87–0.92)	0.97(0.94–1.00)	0.98(0.79–1.21)	0.97(0.78–1.20)
TG	1.13(1.11–1.16)	1.10(1.06–1.13)	1.08(1.02–1.13)	1.07(1.02–1.13)

Hazard ratios (HRs) are provided with 95% confidence intervals

Model 1: adjusted for age, sex, region, BMI, hypertension, diabetes mellitus, the top 10 genetic principal components

Model 2: model1 + income, Townsend deprivation index, smoking, drinking, physical activity (IPAQ), sleep duration, antihypertensive medications usage at baseline and during follow-up, hypoglycemic medications usage at baseline and during follow-up

Model 3: model1 + income, Townsend deprivation index, smoking, drinking, physical activity (IPAQ), sleep duration, antihypertensive medications usage at baseline and during follow-up, hypoglycemic medications usage at baseline and during follow-up, HDL-C, LDL-C, TC, and TG

*HDL-C* high-density lipoprotein cholesterol, *LDL-C* low-density lipoprotein cholesterol, *TC* total cholesterol, *TG* triglycerides, *IPAQ* international physical activity questionnaire, *BMI* body mass index, *HR* hazard ratio

### Global and local genetic correlation

**Fig. 2:**
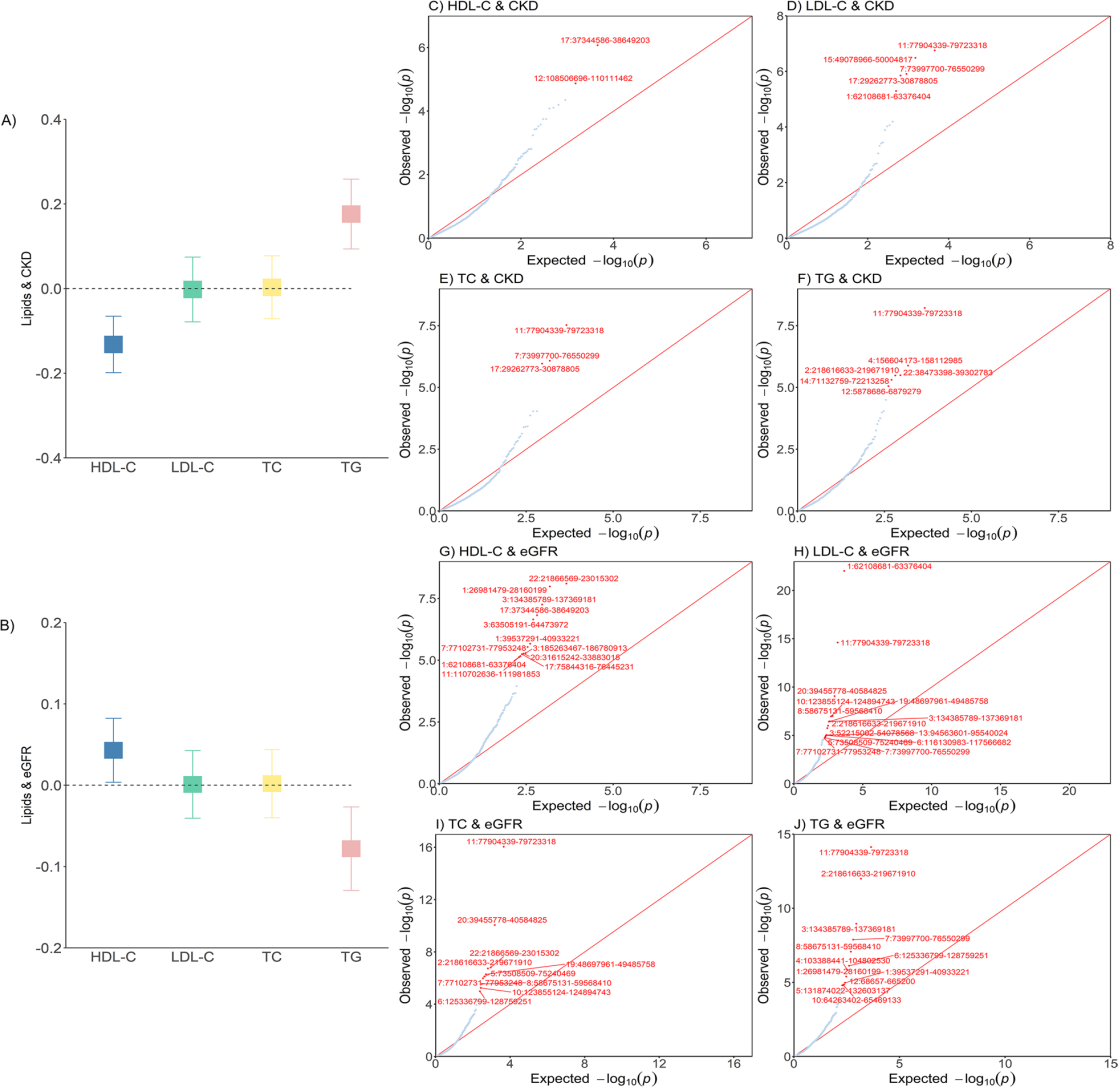
Genome-wide genetic correlation between lipids and chronic kidney disease. The boxes (**A**, **B**) denote the point estimate of the global genetic correlation, and the error bars denote 95% confidence intervals (CI). In the QQ plots (**C–J**), red points represent genomic regions that contribute significant local genetic correlation as estimated by SUPERGNOVA (*P* < 0.05/2353). *HDL-C* high-density lipoprotein cholesterol, *LDL-C* low-density lipoprotein cholesterol, *TC* total cholesterol, *TG* triglycerides, *CKD* chronic kidney disease, *eGFR* estimated glomerular filtration rate

As shown in Fig. 2A, a significant global genetic correlation was observed for CKD with both HDL-C (${r}_{g}$ = -0.132, *P* = 1.00 × 10^–4^) and TG (${r}_{g}$ = 0.176, *P* = 2.66 × 10^–5^). No significant result was found for either LDL-C ( ${r}_{g}$ = − 0.002, *P* = 0.95) or TC ( ${r}_{g}$ = 0.003, *P* = 0.93). For kidney function, a similar pattern of correlation was observed (Fig. 2B).

Partitioning the whole genome into 2,353 LD-independent regions and after correcting for multiple testing (*P* < 2.12 × 10^–5^), we identified twelve genomic regions (HDL-C: 12q23.3-q24.11,17q12; LDL-C: 1P31.3, 7q11.23, 11q14.1, 15q21.1-q21.2, 17q11.2; TC: 7q11.23, 11q14.1, 17q11.2; TG: 2q35, 4q32.1, 11q14.1, 12p13.31, 14q24.2, and 22q13.1) demonstrating a significant local genetic correlation for lipids and CKD (Fig. 2C–F). Notably, chr11:77,904,339–79,723,318 at 11q14.1 was repeatedly identified as a significant region in three of four analyses, harboring *GAB2*, a well-established susceptible locus for both lipids and kidney function and *TENM4*, a previously reported susceptible gene for kidney function. Regarding kidney function, 12 regions were identified for HDL-C with eGFR, 14 for LDL-C, 10 for TC, and 12 for TG (Fig. 2G–J). Detailed information on each region is shown in Fig. 2.

### Cross-trait meta-analysis

**Fig. 3:**
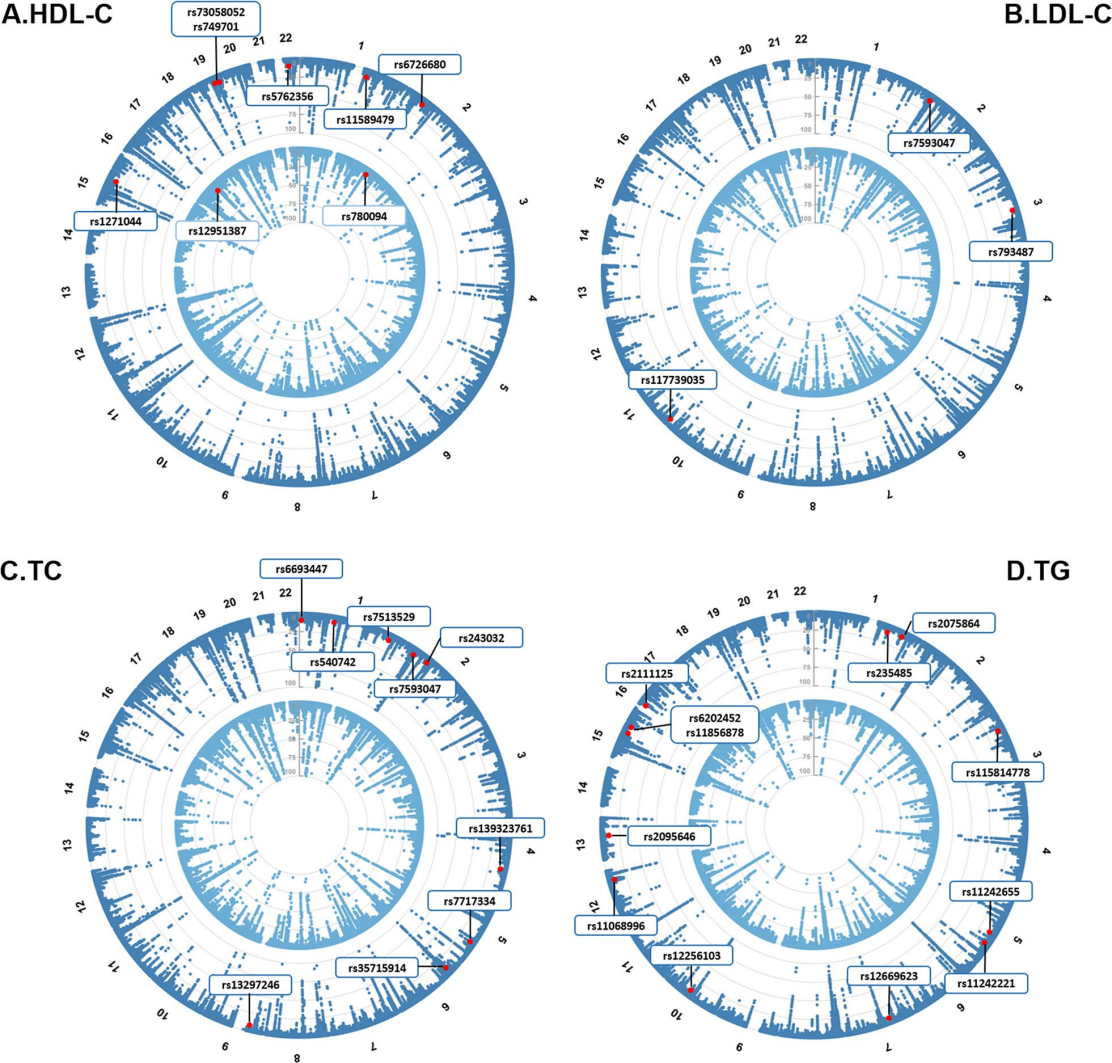
Novel pleiotropic loci between lipids and chronic kidney disease identified from cross-trait meta-analysis. **A** HDL-C and CKD phenotypes; **B** LDL-C and CKD phenotypes; **C** TC and CKD phenotypes; **D** TG and CKD phenotypes. In the circular Manhattan plot, the two circular lanes depict the cross-trait meta-analysis results between lipids traits and CKD phenotypes (blue: eGFR, light blue: CKD). The outermost numbers represent chromosomes 1–22. The red dots represent novel pleiotropic SNPs in the cross-trait meta-analysis (*P*_CPASSOC_ < 5 × 10^–8^, 5 × 10^–8^ < *P*_single-trait_ < 1 × 10^–5^ (in both traits), and were not in LD (*r*^2^ < 0.1) with both single-trait index SNPs). *HDL-C* high-density lipoprotein cholesterol, *LDL-C* low-density lipoprotein cholesterol, *TC* total cholesterol, *TG* triglycerides, *CKD* chronic kidney disease, *eGFR* estimated glomerular filtration rate

We continued to perform pairwise CPASSOC analysis to identify pleiotropic loci (Fig. 3 and Additional file 1: Table S10, S11). In total, we identified 29 independent pleiotropic SNPs shared between lipids and CKD, including 12 loci for HDL-C and CKD, nine loci for LDL-C and CKD, seven loci for TC and CKD, and nine loci for TG and CKD. Among these, we determined two novel SNPs (rs780094 and rs12951387) shared by HDL-C and CKD. SNP rs780094 was mapped to *GCKR*, a pleiotropic gene associated with diabetic and cardiometabolic traits [31, 32], and SNP rs12951387 was mapped to *GGNBP2*, a gene harboring eGFR- and metabolic biomarkers-associated loci [33, 34].

Relaxing the disease status CKD into its underlying physiological measure eGFR, we identified 383 independent pleiotropic SNPs, among which 30 were novel pleiotropic SNPs (6 for HDL-C, 3 for LDL-C, 9 for TC, and 12 for TG).

Detailed annotations of each variant are shown in Additional file 1: Table S12–S19.

### Fine mapping credible-set analysis and colocalization analysis

For all identified pleiotropic SNPs, we determined a 99% credible set of causal SNPs using FM-summary, providing targets for downstream experimental analysis. In particular, we identified only one candidate causal SNP in the 99% credible set for HDL-C and CKD (rs1047891) with a posterior probability of 1.00. With regard to kidney function, more causal variants were identified for lipids and eGFR. Lists of candidate causal SNPs at each pleiotropic locus are shown in Additional file 1: Table S20–S27.

Colocalization analysis was next conducted to determine whether genetic variants driving the association between different traits were the same. We identified 36.4% of shared loci colocalized at the same candidate SNPs (PPH4 > 0.5) for HDL-C-CKD, including two novel shared SNPs (rs780094 and rs12951387). Regarding kidney function, more causal variants were identified (Additional file 1: Table S28, S29).

### Transcriptome-wide association study

We identified multiple independent gene-tissue pairs shared between lipid phenotypes and CKD or kidney function (Additional file 1: Table S30, S31). A total of 36 significant tissue-gene pairs were detected for CKD with at least one lipid phenotype, including seven genes (4 with HDL-C, 3 with LDL-C, 1 with TC, and 6 with TG) mainly enriched in tissues of the nervous, cardiovascular, and reproductive system. Among the 7 TWAS-significant genes, six were previously implicated in lipids and/or kidney function (GWAS Catalog accessed by December 31, 2022), including *PCNX3* associated with HDL-C, *RGS14,* and *CCDC158* associated with eGFR, *MXD3*, *MAP3K11*, and *OVOL1* associated with lipids and eGFR. Regarding kidney function, 892 significant tissue-gene pairs were detected and mainly enriched in tissues of the nervous and cardiovascular systems.

### Bidirectional Mendelian randomization

**Fig. 4:**
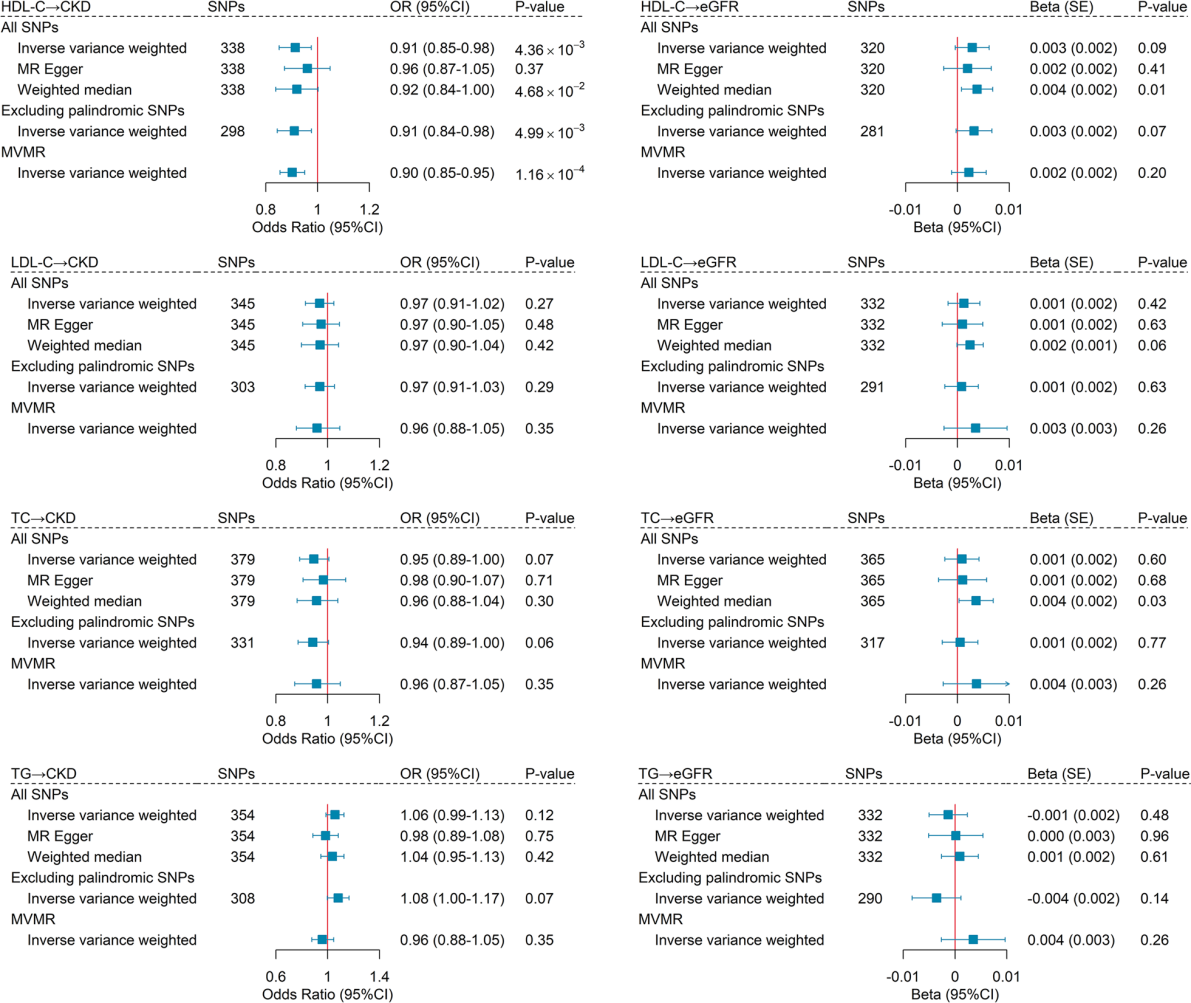
Mendelian randomization analysis between lipids and chronic kidney disease. The boxes denote the point estimate of the causal effects, and the error bars denote 95% confidence intervals (95%CI). MVMR models were adjusted for body mass index, hypertension, type 2 diabetes, HDL-C, LDL-C, TC, and TG. *HDL-C* high-density lipoprotein cholesterol, *LDL-C* low-density lipoprotein cholesterol, *TC* total cholesterol, *TG* triglycerides, *CKD* chronic kidney disease, *eGFR* estimated glomerular filtration rate, *MVMR* multivariable mendelian randomization

We continued to conduct a bidirectional MR to evaluate potential casual associations motivated by the significant shared genetic basis. We only identified a causal relationship of genetically predicted HDL-C (OR_IVW_ = 0.91, 95%CI = 0.85–0.98, *P* = 4.36 × 10^–3^) with the risk of CKD (Fig. 4). The causal estimates remained directionally consistent when using MR-Egger regression (OR = 0.96, 95%CI = 0.87–1.05) or weighted median approach (OR = 0.92, 95%CI = 0.84–1.00). Sensitivity analyses excluding palindromic SNPs (Fig. 4) or using CKD GWAS from the FinnGen consortium (Additional file 1: Table S32) supported the robustness of the results. MVMR generated similar results with even more pronounced magnitude and significance, suggesting an independent causal relationship. No causal association was found for LDL-C, TC, and TG with CKD (LDL-C: OR_IVW_ = 0.97, 95%CI = 0.91–1.02, *P* = 0.27; TC: OR_IVW_ = 0.95, 95%CI = 0.89–1.00, *P* = 0.07; TG: OR_IVW_ = 1.06, 95%CI = 0.99–1.13, *P* = 0.12). For kidney function, no causal association was found between genetically predisposed lipids level and eGFR.

In the reverse-direction MR, genetic predisposition to CKD or eGFR did not seem to causally affect lipids levels. Genetically predicted CKD was only significantly associated with a higher level of TG (beta_IVW_ = 0.018, 95%CI = 0.006–0.031) (Additional file 2: Fig. S1), yet the estimate was not directionally consistent in MR-Egger regression. Regarding kidney function, genetically predisposed eGFR was significantly associated with a lower level of TG (beta_IVW_ = − 0.282, 95%CI = − 0.429–0.136), yet it became nonsignificant in MVMR.

## Discussion

To the best of our knowledge, this is the most comprehensive observational and genetic analysis that systematically investigates the phenotypic association and the shared genetic architecture among circulating lipids, CKD, and kidney function. In both phenotypic and genetic analyses, associations with CKD or kidney function were consistently more evident for HDL-C than for other lipid components. We found a significant shared genetic basis between lipids and CKD or kidney function on several genomic regions, a number of pleiotropic loci, and shared expression-trait pairs. We further identified a pronounced causal effect of HDL-C in the development of CKD.

The intrinsic connection between lipids and CKD, or kidney function reflected by the significant global and local genetic correlations, can be the result of pleiotropy and/or causality. In our downstream MR analysis performed to explore these alternatives, we identified low HDL-C concentration was genetically associated with an increased risk of CKD. Meanwhile, based on the large-scale prospective data, we found a phenotypic association between HDL-C and CKD risk. Consistent with previous MRs [11–13], we further extended the investigation by at least doubling the number of IVs (380 IVs vs. 122 IVs [12]; 380 IVs vs. 84 IVs [11]; 380 IVs vs. 195 IVs [13]). Incorporating additional IVs substantially improved the strength of genetic instruments as well as both the accuracy and precision of MR estimates. Moreover, we detected an independent causal relationship between HDL-C and CKD from MVMR. Although a prior study has additionally corrected the pleiotropic effect of glycated hemoglobin and blood pressure using MVMR, the numbers of incorporated IVs were only 3 ~ 29 [11]. In comparison, our study has distinguished the impact of lipid fractions, T2D, hypertension, and a nonnegligible factor BMI [35], using a substantially enlarged number of IVs (231 ~ 670). Different from HDL-C, we detected no causal effect of LDL-C, TC, and TG on CKD or kidney function. Conversely, findings from Copenhagen studies indicated that high LDL-C was genetically associated with an elevated risk of CKD [2], while a recent two-sample MR study reported the causally protective effect of TC on CKD risk. Besides, another MR analysis integrating results from the UK biobank and the Trøndelag Health Study (HUNT) only found a possible weak effect of higher LDL-C or TG on increased kidney function [15]. The possible explanation for these inconsistent positive findings is the limited sample size and IVs of prior applied genetic data [2, 11–15], resulting in reduced power and accuracy. Additionally, the prior one-sample MR [2] might not have fully considered the exclusion restriction assumption of MR that the instrument–outcome causal pathway must be mediated entirely via the exposure of interest [36], and the study has not tested this assumption by adjusting for measured lipid levels in the association between the weighted genetic scores and outcome risk [37].

Results from cross-trait meta-analyses demonstrated the biological pleiotropy between lipids and CKD or kidney function. Multiple novel shared loci were mapped to genes implicated in alcohol intake (*GIT2* and *HOMER2*) [38, 39], glucose metabolism or diabetes (*FNIP1*, *PFKFB2*, *LINC00393, GCKR*, *RER1*, and *IGF1R*) [40–43], obesity (*GGNBP2*, *ITSN2*, and *FBXL17*) [42, 43], biological processes related to kidney fibrosis or podocyte injury (*CHCHD1*, *PRR12*, *PKP3*, *PPM1B*, *COL8A1,* and *ACTN4*) [44–49], and endothelial function (*ADAM15*, *RAB5A*, and *GAB1*) [50–52], reflecting potential mechanistic pathways linking lipids to CKD. Here we highlight two novel candidate causal variants, rs12951387, and rs780094, shared by HDL-C and CKD. SNP rs12951387 is located in *GGNBP2*, encoding a gametogenetin-binding protein associated with obesity [42, 43, 53]. Obesity could lead to multiorgan ectopic lipid accumulation, characterized by adipocyte hypertrophy, insulin resistance, dysregulation of inflammatory cytokines and adipokines, and stimulation of pro-inflammatory signaling pathways, which could further result in oxidative stress, inflammation, and fibrosis in the kidney, and finally triggers glomeruli and kidney tubule damage [54]. SNP rs780094, located in *GCKR*, is a well-characterized pleiotropic SNP. The T-allele of this SNP is associated with a reduced HDL-C level [10] but a lower diabetes risk [28] and greater kidney function [9], indicating a complex biological mechanism underlying HDL-C and CKD. At the gene-tissue pair level, the multiple genes identified in the nervous and cardiovascular system indicated a biological mechanism through the heart–brain–kidney axis, and more studies are warranted to fully explore these complex mechanisms.

Our findings have important clinical and public health implications. Firstly, HDL-C is a causal protective factor for CKD. Our study indicated the long-term renal-protective effect of managing low HDL-C levels. As clinical evidence showed non-significant improvement of kidney function or reduction CKD incidence via current dyslipidemia treatments primarily targeting cholesteryl ester transfer protein inhibition [4], it is plausible that in addition to HDL-C, other particles, such as sphingosine-1 phosphate, apolipoprotein M, apolipoprotein A-I, or paraoxonase-1, reflecting the functionality of HDL, might also influence kidney health [55–58]. Future research should also explore the effects of these HDL components on CKD onset or kidney function changes. Secondly, our genetic work suggests a common biological mechanism for lipids and CKD. Identifying specific pleiotropic loci and genes regulating common biological pathways may help explore broad-spectrum therapeutic targets that could benefit both precision prevention and treatment of lipid-CKD comorbidity in the future. We hypothesize that aggregating large-scale GWAS to identify common genetic underpinnings may guide new drug development or drug repurposing.

We acknowledge several potential limitations. Firstly, our findings were restricted to individuals of European ancestry. As the relationships between lipids and CKD risk show significant racial differences [13], future studies are expected to extend to other ancestry groups. Secondly, a substantial sample overlap (23.3% in our study) might bias the causal estimations toward observational associations [59]. As the magnitude of bias in IV estimates depends on the *F-statistics* [60], the *F-statistics* were over 289 for lipid instruments and over 56 for CKD instruments in our study, indicating that the potential bias stemming from sample overlap is likely to be minimal. Our sensitivity MR analysis utilizing non-overlapping GWAS data yielded similar results, thereby mitigating the potential for false positive findings. Furthermore, other genome-wide cross-trait analysis approaches demonstrate robustness in dealing with sample overlap [17–19]. Thirdly, two-sample MR using GWAS summary data assumes a linear effect of the exposure on outcome [25]. Recent studies have indicated potential U-shaped relationships between HDL-C levels and the risk as well as the mortality associated with several conditions [61, 62]. Despite the absence of a statistically significant causal association between HDL-C and kidney function in our two-sample MR analysis, future research should leverage individual-level genetic data to elucidate the nature between HDL-C and kidney function. Fourthly, the effects of genetic variables on lipid phenotypes and kidney functions were obtained predominantly from population-based cross-sectional studies and, therefore, may not reflect progression over time. The relationship between the alterations in lipid levels and the change in kidney functions could be further clarified.

## Conclusions

The current study confirms a putative causal effect of HDL-C on CKD through an observational and genetic analysis of European ancestry. Dyslipidemia management targeting low HDL-C levels could help mitigate the long-term burden of CKD. Future studies should consider the potential for non-linear relationships as well as HDL functionality in CKD.

### Supplementary Information

**Additional file 1: Table S1.** Characteristics of genetic instruments of HDL-C. **Table S2.** Characteristics of genetic instruments of LDL-C. **Table S3.** Characteristics of genetic instruments of TC. **Table S4.** Characteristics of genetic instruments of TG. **Table S5.** Characteristics of genetic instruments of chronic kidney disease. **Table S6.** Characteristics of genetic instruments of estimated glomerular filtration rate. **Table S7.** Data sources, sample sizes, number of instruments, and F-statistics. **Table S8.** Baseline Characteristics of UK Biobank participants by chronic kidney disease status during follow-up. **Table S9.** Observational associations between lipids and the risk of chronic kidney disease. **Table S10.** Pleiotropic SNPs identified by cross-trait meta-analysis between lipids and chronic kidney disease. **Table S11.** Pleiotropic SNPs identified by a cross-trait meta-analysis between lipids and estimated glomerular filtration rate. **Table S12.** Detailed annotation of shared SNPs of HDL-C and chronic kidney disease identified from a cross-trait meta-analysis. **Table S13.** Detailed annotation of shared SNPs of LDL-C and chronic kidney disease identified from a cross-trait meta-analysis. **Table S14.** Detailed annotation of shared SNPs of TC and chronic kidney disease identified from a cross-trait meta-analysis. **Table S15.** Detailed annotation of shared SNPs of TG and chronic kidney disease identified from a cross-trait meta-analysis. **Table S16.** Detailed annotation of shared SNPs of HDL-C and estimated glomerular filtration rate identified from a cross-trait meta-analysis. **Table S17.** Detailed annotation of shared SNPs of LDL-C and estimated glomerular filtration rate identified from a cross-trait meta-analysis. **Table S18.** Detailed annotation of shared SNPs of TC and estimated glomerular filtration rate identified from a cross-trait meta-analysis. **Table S19.** Detailed annotation of shared SNPs of TG and estimated glomerular filtration rate identified from a cross-trait meta-analysis. **Table S20.** List of SNPs in the 99% credible set identified from fine-mapping analysis for each CPASSOC-identified locus shared between HDL-C and chronic kidney disease. **Table S21.** List of SNPs in the 99% credible set identified from fine-mapping analysis for each CPASSOC-identified locus shared between LDL-C and chronic kidney disease. **Table S22.** List of SNPs in the 99% credible set identified from fine-mapping analysis for each CPASSOC-identified locus shared between TC and chronic kidney disease. **Table S23.** List of SNPs in the 99% credible set identified from fine-mapping analysis for each CPASSOC-identified locus shared between TG and chronic kidney disease. **Table S24.** List of SNPs in the 99% credible set identified from fine-mapping analysis for each CPASSOC-identified locus shared between HDL-C and estimated glomerular filtration rate. **Table S25.** List of SNPs in the 99% credible set identified from fine-mapping analysis for each CPASSOC-identified locus shared between LDL-C and estimated glomerular filtration rate. **Table S26.** List of SNPs in the 99% credible set identified from fine-mapping analysis for each CPASSOC-identified locus shared between TC and estimated glomerular filtration rate. **Table S27.** List of SNPs in the 99% credible set identified from fine-mapping analysis for each CPASSOC-identified locus shared between TG and estimated glomerular filtration rate. **Table S28.** Results from colocalization analysis for each pleiotropic locus identified from CPASSOC between lipids and chronic kidney disease. **Table S29.** Results from colocalization analysis for each pleiotropic locus identified from CPASSOC between lipids and estimated glomerular filtration rate. **Table S30.** Significant shared transcriptome-wide association analysis results between lipids and chronic kidney disease after Conditional/Joint Analysis. **Table S31.** Significant shared transcriptome-wide association analysis results between lipids and estimated glomerular filtration rate after Conditional/Joint Analysis. **Table S32.** Estimated causal association between HDL-C and chronic kidney disease using non-overlapped data.

**Additional file 2: Figure S1.** Mendelian randomization analysis between chronic kidney disease and lipids traits. The boxes denote the point estimate of the causal effects, and the error bars denote 95% confidence intervals (95%CI). MVMR models were adjusted for body mass index, hypertension, and type 2 diabetes. HDL-C, high-density lipoprotein cholesterol; LDL-C, low-density lipoprotein cholesterol; TC, total cholesterol; TG, triglycerides; CKD, chronic kidney disease; eGFR, estimated glomerular filtration rate; MVMR, multivariable mendelian randomization.

## Data Availability

This research was conducted using the UK Biobank Resource under project 50,538. GWAS summary statistics for lipid phenotypes are available from the Global Lipids Genetics Consortium (http://csg.sph.umich.edu/willer/public/glgc-lipids2021), and GWAS summary statistics for CKD, and eGFR are available through the CKDGen Consortium (https://ckdgen.imbi.uni-freiburg.de/). Genetic correlations: https://github.com/bulik/ldsc. SUPERGNOVA: https://github.com/qlu-lab/SUPERGNOVA. Cross-Phenotype Association analysis: http://hal.case.edu/~xxz10/zhu-web/. PLINK: https://www.cog-genomics.org/plink/1.9/. Fine-mapping: https://github.com/hailianghuang/FM-summary. Colocalization analysis: https://chr1swallace.github.io/coloc/articles/a02_data.html. Transcriptome-wide association study: http://gusevlab.org/projects/fusion/. The usage of each software tool has been described in the Methods section. The analysis code and scripts utilized in the current study are available upon request from the corresponding authors.

## Abbreviations

CKD: Chronic kidney disease
RR: Risk ratio
TG: Triglycerides
HDL-C: High-density lipoprotein cholesterol
LDL-C: Low-density lipoprotein cholesterol
TC: Total cholesterol
MR: Mendelian randomization
GWAS: Genome-wide association studies
eGFR: Estimated glomerular filtration rate
UKB: UK Biobank
ICD-10: International Classification of Diseases, tenth revision
SNP: Single nucleotide polymorphisms
IV: Instrument variable
LD: Linkage disequilibrium
LDSC: Linkage disequilibrium score regression
BMI: Body mass index
CPASSOC: Cross-Phenotype Association analysis
PPH4: Posterior probability of hypothesis 4
TWAS: Transcriptome-wide association study
IVW: Inverse-variance weighted
MVMR: Multivariable Mendelian randomization
HR: Hazard ratio
OR: Odds ratio
T2D: Type 2 diabetes
