# Supplementary figures and images for: Understanding the relationship between circulating lipids and risk of chronic kidney disease: a prospective cohort study and large-scale genetic analyses
Source: J Transl Med. 2023 Sep 27;21:671. doi: 10.1186/s12967-023-04509-5 (PMC10537816; doi:10.1186/s12967-023-04509-5)

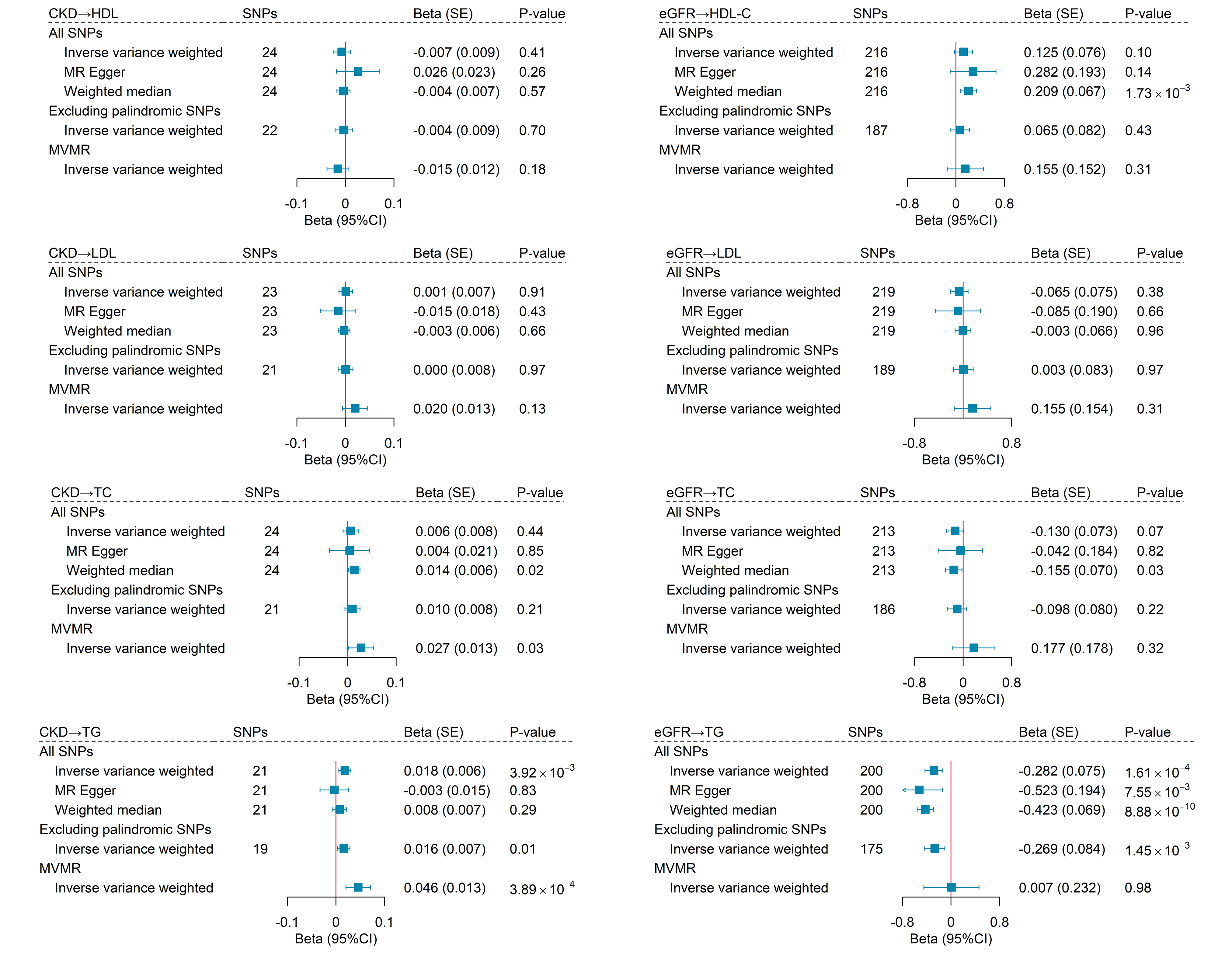

Supplement: Supplementary file 2 — Additional file 2: Figure S1. Mendelian randomization analysis between chronic kidney disease and lipids traits. The boxes denote the point estimate of the causal effects, and the error bars denote 95% confidence intervals (95%CI). MVMR models were adjusted for body mass index, hypertension, and type 2 diabetes. HDL-C, high-density lipoprotein cholesterol; LDL-C, low-density lipoprotein cholesterol; TC, total cholesterol; TG, triglycerides; CKD, chronic kidney disease; eGFR, estimated glomerular filtration rate; MVMR, multivariable mendelian randomization. [file 12967_2023_4509_MOESM2_ESM.tif]
